# Mortality differences among patients with in‐hospital ST‐elevation myocardial infarction

**DOI:** 10.1002/clc.23480

**Published:** 2020-11-07

**Authors:** Negeen Shahandeh, Xuming Dai, Brian Jaski, Ravi Dave, Alice Jacobs, Ali Denktas, Glenn Levine, Daniela Markovic, Sidney C. Smith, Marcella Calfon Press

**Affiliations:** ^1^ University of California Los Angeles Los Angeles California USA; ^2^ University of North Carolina Chapel Hill North Carolina USA; ^3^ Sharp Memorial Hospital San Diego San Diego California USA; ^4^ Boston University Medical Center Boston Massachusetts USA; ^5^ The Michael E. DeBakey VA Medical Center Houston Texas USA; ^6^ Baylor College of Medicine Houston Texas USA

**Keywords:** acute coronary syndrome, ischemic heart disease, myocardial infarction, percutaneous coronary intervention

## Abstract

**Background:**

In‐hospital ST‐elevation myocardial infarction (STEMI) is associated with a higher mortality rate than out‐of‐hospital STEMI. Quality measures and universal protocols for treatment of in‐hospital STEMI do not exist, likely contributing to delays in recognition and treatment.

**Hypothesis:**

To analyze differences in mortality among three subsets of patients who develop in‐hospital STEMI.

**Methods:**

This was a multicenter, retrospective observational study of patients who developed in‐hospital STEMI at six United States medical centers between 2008 and 2017. Patients were stratified into three groups: (1) cardiac, (2) periprocedure, or (3) noncardiac/nonpostprocedure. Outcomes examined include time from electrocardiogram (ECG) acquisition to cardiac catheterization lab arrival (ECG‐to‐CCL) and survival to discharge.

**Results:**

We identified 184 patients with in‐hospital STEMI (mean age 68.7 years, 58.7% male). Group 1 (cardiac) patients had a shorter average ECG‐to‐CCL time (69 minutes) than group 2 (periprocedure, 215 minutes) and group 3 (noncardiac/nonpostprocedure, 199 minutes). Compared to group 1, survival to discharge was lower for group 2 (OR 0.33, *P* = .102) and group 3 (OR 0.20, *P* = .016). After adjusting for prespecified covariates, the relationship between group and survival showed a similar trend but did not reach statistical significance.

**Conclusions:**

Patients who develop in‐hospital STEMI in the context of a preceding procedure or noncardiac illness appear to have longer reperfusion times and higher in‐hospital mortality than patients admitted with cardiac diagnoses. Larger studies are warranted to further investigate these observations. Health systems should place an increased emphasis on developing quality metrics and implementing quality improvement initiatives to improve outcomes for in‐hospital STEMI.

**Abbreviations**
CABG: coronary artery bypass grafting
ECG: electrocardiogram
ICD: international classification of diseases
OR: odds ratio
PCI: percutaneous coronary intervention
STEMI: ST‐elevation myocardial infarction

## 1 INTRODUCTION

In‐hospital ST‐elevation myocardial infarction (STEMI) is a unique clinical entity encompassing a more heterogeneous patient population and associated with a higher mortality rate than out‐of‐hospital STEMI.^1^ The true incidence of in‐hospital STEMI is unclear due in the past to the lack of a standardized definition and the exclusion of these patients from clinical trials, however it is estimated at 18 to 34 per 100 000 adult hospitalizations based on the available literature.^1^ Prior retrospective studies show that patients who develop in‐hospital STEMI are older, more frequently female, have higher in‐hospital mortality, and less frequently undergo cardiac catheterization than patients who present with out‐of‐hospital STEMI. Mortality of in‐hospital STEMI is 3 to 10‐fold greater than out‐of‐hospital STEMI.^2^, ^3^, ^4^ An integrated system of care, along with national quality initiatives such as the American college of cardiology door to balloon alliance and the American heart association's mission: Lifeline, have resulted in expedient reperfusion times and decreased mortality for patients who develop out‐of‐hospital STEMI.^5^, ^6^, ^7^ Conversely, for patients who develop STEMI while admitted to the hospital, there are often barriers to diagnosis and management. Equivalent quality measures and universal protocols for treatment of in‐hospital STEMI do not exist, likely contributing to delays in recognition and treatment.

Recently, a national cohort of clinicians and researchers have formed the In‐Hospital STEMI Quality Improvement Project, a group dedicated to increasing awareness of and improving outcomes for patients with in‐hospital STEMI. In a recent Special Communication published in JAMA Cardiology, this group proposed a standardized definition of in‐hospital STEMI and identified three clinically distinct groups within this cohort.^1^ However, the value of this classification has not yet been determined. The aim of the study presented here is to evaluate the usefulness of this standardized classification and to analyze outcomes with respect to these groups.

## 2 METHODS

**FIGURE 1:**
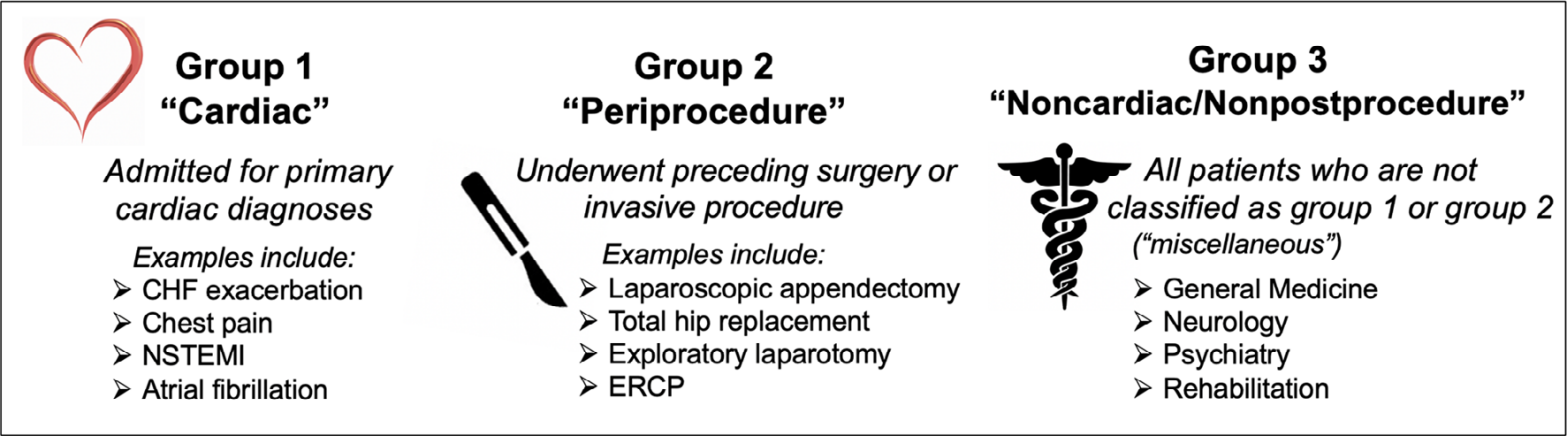
In‐Hospital STEMI Subgroup DefinitionsPatients with in‐hospital STEMI can be classified into three groups, as defined recently in the literature: 1) cardiac patients (admitted for primary cardiac diagnoses), 2) periprocedure patients (those who underwent a preceding surgery or invasive procedure during the index hospitalization), or 3) noncardiac/nonpostprocedure patients (“miscellaneous”)^1^. STEMI, ST‐elevation myocardial infarction

This was a retrospective, multicenter observational study of patients who developed in‐hospital STEMI. The study was approved by the institutional review board at each participating center. Cases were identified from hospital discharges between 2008 to 2017 using ICD‐9‐CM and ICD‐10‐CM diagnosis codes for STEMI (410.x and I21). All hospitalizations with these diagnosis codes present on admission were excluded. Remaining cases were subsequently reviewed by physicians at each site to verify the occurrence of STEMI during the index hospitalization. In‐hospital STEMI in this study was defined according to the standardized definition proposed by the In‐Hospital STEMI Quality Improvement Project Special Communication published in February 1, 2018.^1^ All cases were classified into one of the three clinical groups as defined by the In‐Hospital STEMI Quality Improvement Project and depicted in Figure 1, based on the reason for admission and preceding surgical or invasive procedure during the index hospitalization. Group 1 (“cardiac”) encompasses patients admitted with a primary cardiac diagnosis or those who have undergone a recent cardiac procedure such as percutaneous coronary intervention (PCI) or coronary artery bypass graft surgery (CABG). Group 2 (“periprocedure”) comprises patients who developed STEMI during or after noncardiac procedures within the same index hospitalization. All other patients not included in groups 1 or 2 are assigned to group 3 (“noncardiac/nonpostprocedure”). Included in this group are all patients admitted for neurological, psychiatric, and medical reasons as well as patients admitted to surgical services (preoperatively).^1^

Primary endpoints analyzed were time from electrocardiogram (ECG) acquisition to arrival in the cardiac catheterization lab (ECG‐to‐CCL) and survival to discharge. Clinical and demographic characteristics were compared across the three groups using the Chi‐Square/Fisher's test, as appropriate. Due to a large number of missing data points for ECG‐to‐CCL time, examination of this outcome was limited to descriptive analysis. We evaluated the relationship between each group and survival to discharge using a logistic regression model before and after adjusting for prespecified covariates. Our study had 67 events which allowed us to reliably control for at most six potential confounders (history of diabetes mellitus, history of hypertension, history of chronic kidney disease, gender, ECG trigger, and whether or not the patient underwent cardiac catheterization). These variables were deemed to be the most important factors for mortality and were prespecified as potential confounders. When age was forced to the final model, the results were essentially unchanged, indicating that age was not an important factor for mortality in this patient population. The final model was selected using the backwards procedure for variable selection and liberal *P* < .15 as the retention criterion (history of hypertension and whether or not the patient underwent cardiac catheterization were the only covariates that met retention criteria, therefore the others were removed from the final model). Sample size calculation was based on differences in mortality between groups 2 and 3 vs group 1 based on the logistic model. Based on the current sample sizes, there was 80% power to confirm differences in mortality of 31% or greater between the groups, assuming that mortality in group 1 was 14% using an alpha level of 0.05 for each comparison.

## 3 RESULTS

**FIGURE 2:**
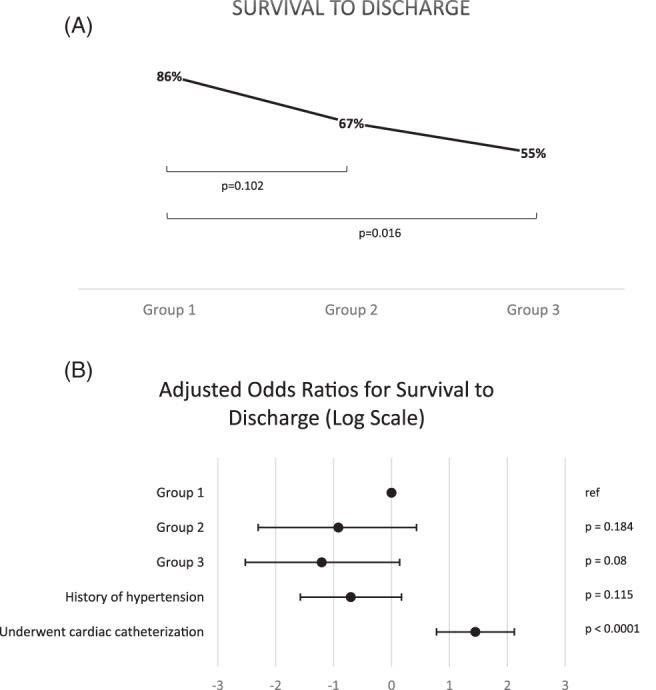
A, Unadjusted analysis ‐ survival to dischargeGroup 1 had the highest survival to discharge (86%) of the three cohorts, followed by group 2 (67%). Group 3 patients had the worst survival to discharge (55%), which was significantly lower when compared to group 1 (*P* = .016). B, Adjusted analysis ‐ survival to dischargeAfter a logistic regression analysis, there was a trend toward lower adjusted odds of survival for groups 2 and 3 compared to group 1, but findings did not reach statistical significance. Of the covariates analyzed, undergoing cardiac catheterization was found to be associated with significantly greater odds of survival to discharge (*P* < .0001)

Between 2008 to 2017, there were 184 cases of confirmed in‐hospital STEMI across six medical centers in the United States. The mean age of patients was 68.7 years and 58.7% were male. ECG acquisition was triggered by patient‐reported cardiac symptoms in 47% of patients and by staff‐observed changes in clinical status or telemetry abnormalities in 53% of patients. One hundred and twenty‐one patients (66%) underwent cardiac catheterization. A culprit lesion was identified in 89% of these 121 patients, and 91 patients (75% of those that underwent cardiac catheterization) also underwent PCI. Of the 25% that did not undergo PCI, the primary reasons cited were the absence of a culprit lesion or the identification of a lesion that was not amenable to PCI. Data for time of ECG acquisition and cardiac catheterization lab arrival was available for 73 (60%) patients. Among these patients, the average time from ECG acquisition to arrival in the cardiac catheterization lab (ECG‐to‐CCL time) was 200 minutes (median 91 minutes, interquartile range 53‐215 minutes). Survival to discharge (Figure 2A) and ECG‐to‐CCL time were further analyzed within each group and results are reported below. Of the 184 cases, 21 met criteria for group 1 (“cardiac”), 78 for group 2 (“periprocedure”), and 85 for group 3 (“noncardiac/nonpostprocedure”). When stratified by group, there was no significant difference in age (*P* = .86), history of coronary artery disease (*P* = .35), hypertension (*P* = .43), diabetes mellitus (*P* = .61) or hyperlipidemia (*P* = .89) between the three groups (Table 1). A similar proportion of patients in each group were on background medical therapy for coronary artery disease, however patients in groups 2 and 3 were significantly more likely than those in group 1 to have had interruption of medical therapy during the hospitalization (Table 1). Notably, the periprocedure and noncardiac/nonpostprocedure groups were comprised of significantly more women than in the cardiac group (41% and 48% vs 14%).

**TABLE 1:** Patient characteristics

	Group 1(n = 21)	Group 2(n = 78)	Group 3(n = 85)	*P* value
Mean age (years)	69.5	68.1	69.1	.86
Female	14%	41%	48%	.018
Medical regimen				
Antiplatelet therapy Dual antiplatelet therapy Statin Beta blocker	14 (67%) 3 (14%) 12 (57%) 10 (48%)	49 (63%) 13 (17%) 38 (49%) 40 (51%)	41 (48%) 8 (9%) 40 (47%) 44 (52%)	.10 .38 .71 .94
Interruption of cardiac medications				
Antiplatelet Statin Beta blocker	0 (0%) 0 (0%) 0 (0%)	23 (29%) 13 (16%) 13 (17%)	10 (12%) 8 (9%) 14 (16%)	.0007 .0832 .101
History of CAD/MI	11 (52%)	30 (38%)	30 (35%)	.35
Hypertension	16 (76%)	59 (76%)	71 (84%)	.43
Diabetes mellitus	9 (43%)	29 (37%)	38 (45%)	.61
Hyperlipidemia	14 (67%)	51 (65%)	53 (62%)	.89
History of tobacco use	16 (76%)	38 (49%)	38 (45%)	.03
Telemetry	19 (90%)	44 (56%)	42 (49%)	.003
Underwent cardiac catheterization	18 (86%)	55 (70%)	48 (56%)	.02
Culprit lesion	18 (86%)	44 (80%)	46 (96%)	.01
Underwent PCI	15 (71%)	39 (50%)	37 (44%)	.07

Abbreviation: PCI, percutaneous coronary intervention.

### 3.1 Group 1 ‐ “cardiac”

**FIGURE 3:**
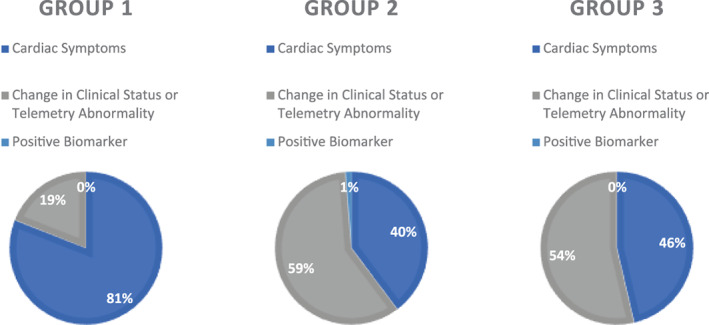
ECG Triggers by groupThe initial trigger for ECG acquisition was significantly different between cardiac (group 1) patients and noncardiac (groups 2 and 3) patients (*P* = .0051). ECGs were more frequently obtained due to observed telemetry abnormalities or changes in clinical status among the noncardiac patients, whereas chest pain was the more frequent trigger for ECG acquisition in cardiac patients. Only one patient in the study had an ECG triggered by positive biomarker. ECG, electrocardiogram

The mean age of the cardiac patients was 69.5 years. These patients were more commonly male gender (86%) than the noncardiac groups and ECG acquisition was more often triggered by reported cardiac symptoms rather than change in clinical status or telemetry abnormalities (Figure 3). The majority of these patients were admitted to cardiac primary services and monitored on telemetry at the time of the STEMI event (Table 1). Of the 21 patients, 18 (86%) underwent cardiac catheterization and 15 (71%) underwent PCI. All of the patients that underwent cardiac catheterization were found to have culprit lesions. The average ECG‐to‐CCL time was 69 minutes, though notably these data were only available for four patients in this cohort. Cardiac patients had an 86% survival to discharge.

### 3.2 Group 2 ‐ “periprocedure”

The mean age in the periprocedure group was 68.1 years. These patients were more likely than cardiac patients to be female gender (41% vs 14%) and ECGs were more likely to be acquired due to a change in clinical status or observed telemetry abnormalities as opposed to reported symptoms (Figure 3). Fewer of these patients underwent cardiac catheterization than in the group 1 cohort (55/78, 71%), however 80% (44/55) of those that underwent coronary angiography had a culprit lesion, and 71% (39/55) were treated with PCI. The average ECG‐to‐CCL time in this cohort was 215 minutes and survival to discharge was 67%.

### 3.3 Group 3 ‐ “noncardiac/nonpostprocedure”

The remaining 85 cases were categorized as part of the group 3 cohort. Mean age among this cohort was 69.1 years. Similar to the group 2 patients, these patients were also more likely to be female gender than cardiac patients (48% vs 14%) and more commonly had ECGs obtained for a change in clinical status or telemetry abnormality rather than cardiac symptoms (Figure 3). Average ECG‐to‐CCL time was 199 minutes and survival to discharge was 54%, the lowest of the three groups. These patients were also less likely to undergo cardiac catheterization than the group 1 cardiac patients. Only 56% (48/85) of this cohort were taken to the CCL, as opposed to 86% in the group 1 cohort. Of those patients that underwent coronary angiography, however, 96% (46/48) were found to have a culprit lesion and 77% (37/48) subsequently underwent PCI. In both group 2 and group 3, the decision to forego cardiac catheterization was frequently due to a higher incidence of contraindication and/or clinical instability.

### 3.4 Survival to discharge

In the unadjusted analysis, the odds of survival was significantly lower for group 3 compared to group 1 (OR 0.20, *P* = .016). The unadjusted odds of survival for group 2 was also lower than group 1, but this association did not reach statistical significance (OR 0.33, *P* = .102). In multivariate analysis, only cardiac catheterization and hypertension were significantly associated with survival. In particular, whether or not a patient underwent cardiac catheterization, regardless of the anatomy, was associated with a significantly greater odds of survival to discharge (OR 4.25, *P* < .001). History of hypertension was associated with decreased survival to discharge, but this was not statistically significant (OR 0.5, *P* = .12). After adjustment for covariates, the relationship between group and survival showed a similar trend, although the differences across groups did not reach statistical significance (Figure 2B).

## 4 DISCUSSION

In‐hospital STEMI, while far less common than out‐of‐hospital STEMI, is associated with delayed reperfusion and poorer outcomes.^1^ Admitted patients are frequently not on cardiology services, less likely to have typical symptoms suggestive of acute MI, and more likely to have comorbid conditions which may serve as contraindications to invasive diagnostic testing, including coronary angiography. These factors contribute to delayed recognition, triage, and treatment of in‐hospital STEMI.^1^ The cohort of patients who develop in‐hospital STEMI consists of a heterogeneous population with various admission diagnoses and varying complexity of illness. This study highlights the differences in outcomes among three distinct subsets of patients who develop STEMI while admitted to the hospital: cardiac, periprocedure, and noncardiac/nonpostprocedure. To our knowledge, this is the first multicenter study to stratify cases of in‐hospital STEMI into more discrete, homogeneous groups.

Although an overall in‐hospital mortality of 36% presented in this case series was comparable to published mortality rates, we found that this was driven primarily by higher mortality among the noncardiac patients (33%‐46%).^1^ Mortality for cardiac patients in this study more closely resembled reported rates of out‐of‐hospital STEMI cases.^8^ Furthermore, the noncardiac patients in our study had longer ECG‐to‐CCL times than cardiac patients, although a significant limitation is the extent of missing data and the small sample size, particularly for our cohort of cardiac patients. Nonetheless, our findings were consistent with those reported in single center studies by Garberich et al and Dai et al, which also found that patients admitted with noncardiac reasons had longer reperfusion times and higher mortality than those admitted for cardiac reasons.^3^, ^9^

Prior studies have also found that delays in time from ECG acquisition to coronary angiography contribute to the delays in reperfusion seen for in‐hospital STEMI.^3^ This is frequently due to nonsystem delays, including recognition by staff of the need to activate existing STEMI systems, time required to stabilize patients, and time to risk stratify patients for reperfusion therapy. In a 2013 retrospective analysis of 48 in‐hospital STEMI cases, Dai et al found that the primary sources of delays in reperfusion were prolonged times from initial event to ECG acquisition and from ECG acquisition to coronary angiography.^3^ Garberich et al found that patients who develop STEMI after admission to the hospital had longer ECG‐to‐balloon times than those who present through the emergency department.^9^ One potential explanation for these findings is that healthcare providers and ancillary staff on noncardiac floors are not trained in the recognition of STEMI or ECG interpretation and have limited knowledge of how to activate existing STEMI pathways in the hospital. These gaps in training, coupled with the often atypical presentations of in‐hospital STEMI, likely contribute significantly to the long ECG‐to‐CCL times we found in this study and may represent opportunities for systems improvement.^4^

The data presented in this study also provide insight into risk factors that may be associated with poorer outcomes among patients who develop in‐hospital STEMI. These include a preceding surgery or invasive procedure, admission for a noncardiac diagnosis, and admission to a noncardiac primary service. Furthermore, the noncardiac patients (groups 2 and 3) in our study were significantly more likely to have had interruption of their antiplatelet regimen during the hospitalization than the cardiac patients, which may provide an additional explanation for poorer outcomes among these patients. Despite the differences between the three groups, 89% of all patients had a culprit lesion identified on coronary angiography, and greater than 70% of all patients taken to the cardiac catheterization laboratory underwent PCI, suggesting that acute coronary syndrome remains the most likely cause of in‐hospital STEMI, even among noncardiac patients. Furthermore, despite delays in reperfusion, undergoing cardiac catheterization, regardless of coronary anatomy, was associated with increased likelihood of survival to discharge (adjusted OR 4.25, *P* < .0001). This relationship is likely confounded by selection bias, as the ability to undergo the procedure selected lower risk patients and excluded those with higher probability of in‐hospital mortality. Indeed, the most commonly cited reasons for not undergoing cardiac catheterization were clinical instability and the presence of contraindications to the procedure, such as bleeding. Although a causal relationship cannot be established without randomization, the observation that inability to undergo cardiac catheterization is associated with poorer hospital survival may be useful for prognostication when discussing goals of care for critically ill patients who suffer in‐hospital STEMI. Another notable observation was the high incidence of ECG acquisition triggered by changes in clinical status and abnormalities noted on telemetry, particularly among noncardiac patients, suggesting a possible role for the use of telemetry monitoring in the early detection of in‐hospital STEMI.

Previous studies have also shown that patients who have in‐hospital STEMI are more likely to be of female gender.^2^ In our study, there was a higher prevalence of female patients in the noncardiac groups as compared to the cardiac group. A significant limitation to the study, however, is the uneven distribution of cases among the three groups, most notably the small size of the group 1 cohort. Due to the limited data available for cardiac patients, observations noted in this cohort may not accurately represent the true estimates or outcomes of the national population. Furthermore, the majority of patients in the group 1 cohort within our study were treated at a Veterans' Affairs Medical Center, which may bias the gender distribution resulting in more male patients within this cohort, further limiting generalizability of the findings to a broader population. Despite this potential bias, one possible explanation for the overwhelming prevalence of male patients in the group 1 cohort is that women are more likely to present with atypical cardiac symptoms and be inappropriately triaged to noncardiac services upon admission.^10^ Regardless, the majority of patients across all groups was male. This may suggest that in‐hospital STEMI, like out‐of‐hospital STEMI, more commonly occurs in male patients or that in‐hospital STEMI is underdiagnosed in female patients, potentially due to differences in clinical presentation among women. Another possible explanation is that women who develop in‐hospital STEMI are more likely to do so in the setting of acute noncardiac illness or after invasive procedures than men are. There was no significant difference in survival when adjusting for gender in this study, though the study was not powered to observe differences in gender due to the low number of female patients in the cohort. Future studies with a larger cohort of both female patients and cardiac patients are needed to identify the potential association of female gender with development of “noncardiac” in‐hospital STEMI, as well as the effect of gender on mortality.

There are several additional limitations to the current study. This was a retrospective analysis with reliance on ICD‐9 and ICD‐10 codes to identify cases meeting inclusion criteria. Therefore, there is a possibility that cases were inadvertently excluded if patients died prior to diagnosis, if the physician did not document the appropriate diagnoses, or if there was incomplete or inaccurate coding. For those patients that had ECG and biomarker findings suggestive of STEMI that did not undergo cardiac catheterization (63/184), we cannot exclude the possibility that these findings were due to STEMI mimickers such as stress‐induced cardiomyopathy or pericarditis. Furthermore, we did not collect data regarding cause of death, therefore it is unknown whether in‐hospital mortality was related to the STEMI event. The lack of left ventricular systolic function in our analysis is also a limitation, given that this is a factor that may influence patient outcomes.

Our results demonstrated that differences in survival were 24% between groups 1 and 3 and 17% between groups 1 and 2, but these differences did not reach statistical significance. In order to confirm these differences with 80% power at an alpha level of 0.05, a larger sample size is needed (35 patients in group 1, 295 patients in group 2, and 167 in group 3). Therefore, the lack of statistical significance is likely due to small sample sizes. Additional studies are warranted to confirm our findings, to further investigate variables associated with the development of in‐hospital STEMI, and to elucidate the etiologies of prolonged reperfusion times. Despite delays in reperfusion, the use of PCI has been associated with higher rates of survival in these patients.^7^ Prospective data are also needed to further assess the impact of PCI on mortality. Quality improvement initiatives aimed at reducing these delays are being developed and implemented at medical centers across the country, and data from one center showed that the implementation of a standardized protocol for inpatient STEMI management reduced reperfusion times and improved mortality rates within 1 year.^9^ Prospective data from more medical centers is needed to assess the efficacy of such initiatives and to facilitate the development of national quality measures similar to those in place for out‐of‐hospital STEMI. Another valuable goal of future research would be to develop and validate a risk score to identify patients at the highest risk of developing in‐hospital STEMI upon admission so that targeted interventions for prevention and early diagnosis can be implemented.

## 5 CONCLUSION

The classification system of in‐hospital STEMI used in this study identifies three clinically distinguishable patient populations, which is helpful for standardized reporting in future studies of in‐hospital STEMI. Of patients that develop in‐hospital STEMI, those who are categorized as periprocedure (group 2) or noncardiac/nonpostprocedure (group 3) appeared to have longer delays in reperfusion and higher in‐hospital mortality than patients admitted with cardiac complaints (group 1). Furthermore, inability to undergo cardiac catheterization after the development of in‐hospital STEMI is associated with increased in‐hospital mortality. These observations warrant further investigation with a larger sample size. Health systems should place a heightened emphasis on the development of quality improvement metrics and initiatives in order to improve clinical outcomes for patients who suffer in‐hospital STEMI.

## CONFLICT OF INTEREST

The authors declare no potential conflict of interest.

## Data Availability

The data that support the findings of this study are available from the corresponding author upon reasonable request.

## References

[1] Levine GN , Dai X , Henry TD , et al. In‐hospital ST‐segment elevation myocardial infarction: improving diagnosis, triage, and treatment. JAMA Cardiol. 2018;3:21‐531. 10.1001/jamacardio.2017.5356. 29466558

[2] Kaul P , Federspiel JJ , Dai X , et al. Association of Inpatient vs outpatient onset of ST‐elevation myocardial infarction with treatment and clinical outcomes. JAMA. 2014;312(19):1999‐2007. 10.1001/jama.2014.15236. 25399275 PMC4266685

[3] Dai X , Bumgarner J , Spangler A , Meredith D , Smith SC , Stouffer GA . Acute ST‐elevation myocardial infarction in patients hospitalized for noncardiac conditions. J Am Heart Assoc. 2013;2:e000004 10.1161/JAHA.113.000004. 23557748 PMC3647284

[4] Dai X , Kaul P , Smith SC Jr , Stouffer GA . Predictors, treatment, and outcomes of STEMI occurring in hospitalized patients. Nat Rev Cardiol. 2015;13:148. 26525542 10.1038/nrcardio.2015.165

[5] Krumholz HM , Bradley EH , Nallamothu BK , et al. A campaign to improve the timeliness of primary percutaneous coronary intervention. Door‐to‐Balloon: An Alliance for Quality JACC Cardiovasc Interv. 2008;1(1):97‐104. 19393152 10.1016/j.jcin.2007.10.006

[6] Bradley EH , Nallamothu BK , Herrin J , et al. National efforts to improve door‐to‐balloon time results from the door‐to‐balloon Alliance. J Am Coll Cardiol. 2009;54(25):2423‐2429. 20082933 10.1016/j.jacc.2009.11.003

[7] Jacobs AK , Antman EM , Ellrodt G , et al. Recommendation to develop strategies to increase the number of ST‐segment‐elevation myocardial infarction patients with timely access to primary percutaneous coronary intervention. Circulation. 2006;113(17):2152‐2163. 16569790 10.1161/CIRCULATIONAHA.106.174477

[8] Mcnamara RL , Wang Y , Herrin J , et al. Effect of door‐to‐balloon time on mortality in patients with ST‐segment elevation myocardial infarction. J Am Coll Cardiol. 2006;47(11):2180‐2186. 16750682 10.1016/j.jacc.2005.12.072

[9] Garberich RF , Traverse JH , Claussen MT , et al. ST‐elevation myocardial infarction diagnosed after hospital admission. Circulation. 2014;129(11):1225‐1232. 24389237 10.1161/CIRCULATIONAHA.113.005568

[10] Canto JG , Rogers WJ , Goldberg RJ , et al. Association of age and sex with myocardial infarction symptom presentation and in‐hospital mortality. JAMA. 2012;307(8):813‐822. 22357832 10.1001/jama.2012.199 PMC4494682

